# Oncology clinical trials in the time of COVID-19: how a pandemic can revolutionize patients’ care

**DOI:** 10.2217/fon-2020-0364

**Published:** 2020-05-14

**Authors:** Francesco Massari, Veronica Mollica, Stefania Salvagni, Michele Tognetto, Andrea Ardizzoni

**Affiliations:** ^1^Division of Oncology, S-Orsola-Malpighi Hospital, Bologna, Italy

**Keywords:** clinical trials, coronavirus, COVID-19, oncology, patient benefit, treatment decisions

According to the WHO, as of 26 April 2020, there are 2,804,796 confirmed cases of coronavirus disease 2019 (COVID-19) and 193,722 confirmed death worldwide [1]. The rapid spread of COVID-19 pandemic is forcing the oncological community to revise treatment protocols, visit schedules and most of all, treatment decisions [2–4].

Many countries worldwide are on lockdown to limit the COVID-19 outbreak. Hospital visits endanger patients as they need to leave their homes and attend crowded places with high risk of infection [5,6]. Indeed, recent studies show that cancer patients present a higher risk of COVID-19 infection (odds ratio: 2.31; 95% Cl: 1.89–3.02) and of severe events from COVID-19 [5,6]. Some adopted containment measures require to postpone not necessary visits or to conduct them through telemedicine [2,3].

One of the contingency measures adopted by most pharmaceutical companies during this emergency is to stop patients’ screening and enrollment in clinical trials [7]. The rationale of this decision lies on the expected difficulty for patients to attend all screening procedures, treatment visits, laboratory exams, study assessments and on the increased risk of patients drop out and adverse events occurrence.

The necessary measures adopted to contain the spread of the COVID-19 infection could have a drastic impact on protocol procedures that are often cumbersome and would require frequent patients’ presence in the hospital. In some cases, the decision to stop trial enrollment is made by study sponsors in a very rapid time frame, thus forcing investigators to withdraw a therapeutic option already proposed and explained to patients and to disrupt their hopes and trust in the medical staff. In this confusing environment, clinicians may not know how to proceed since, one day you may be able to propose an experimental protocol and the next day you may be facing a halt to screening or enrollment.

The main concern of medical investigators is to provide what is best for our patients, taking into account all available therapeutic options, including those deriving from the enrollment into a clinical trial and possible risks connected to the experimental treatment together with patients’ expectations. Furthermore, it is well known that patients enrolled into clinical trials have a better outcome due to better treatment options and best clinical practice procedures. In the ethical and medical decision process, there are two heavy weights on the scale: on one hand, the therapeutic benefits we want to provide for our patients, especially if other treatment strategies are not available or not clinically competing; on the other hand, there is the significant risk of infection that these fragile patients are subjected to. In addition, many oncological treatments, experimental or not, expose patients to immunosuppression, which is a risk factor for worse outcomes from COVID-19 [5]. The decision to stop patients’ enrollment is another of the worrisome consequences of the spread of COVID-19 since it narrows the therapeutic strategies at our disposal. Hospitals are putting in place all containment measures possible, such as dedicated wards in the hospital for COVID-19 patients, personal protective equipment for patients and healthcare workers, limitation of patients’ relatives’ hospital access.

This process also sheds light on the position of pharmaceutical companies toward experimental treatments: in normal times these are therapeutic chances, but they are addressed as potentially dangerous in this time of sanitary emergency. Furthermore, insurance issues can arise because suspension of protocols could invalidate insurance clauses thus exposing investigators to legal repercussions.

If experimental treatments are considered by pharmaceutical companies, to be not as necessary as approved therapies during this particular time, why should they be any different and be presented as the best therapeutic options in other moments?

If patients are able to responsibly attend visits and procedures and are willing to take the risk for themselves because they and their clinicians consider the experimental treatment to give a fighting chance in their cancer struggle, why should we, as investigators, refrain from giving our patients this opportunity? A thorough discussion of experimental treatment expected benefits and risks, protocol requirements in terms of screening procedures, laboratory and radiological assessments and alternative therapeutic options should be carried on with the patient to consider if he is able and willing to proceed with the clinical study.

We must also consider some of the most important ethical principles such as patient autonomy and beneficence? Basic ethical principles in research involving human subjects are respect of persons, beneficence and justice [8]. Patients’ beneficence consists of respecting their decisions and protecting them from harm, while securing their well-being and maximizing possible benefits [8]. The principle of respect acknowledges patients’ autonomy and implies to give weight to autonomous persons’ opinions and choices [8]. The risk/benefit ratio, that takes into account exposure to COVID-19 and possible benefits from the experimental treatment proposed should be evaluated in concordance with the patient under the clinical guidance of the oncologist.

Cancer outcome still scares patients more than COVID-19 infection. Is the fear of having minor protocol deviations over-riding the main aim of clinical trial research, which is to give our patients a further therapeutic chance? Protocol deviations are expected during the COVID-19 emergency and should be assessed and reported by good clinical practice inspectors, but should not be considered as noncompliance with the protocol when the best interest and safety of the participant is protected [7].

Are pharmaceutical companies always taking decisions in our patients’ best interest? Will COVID-19 take out people lives and patients’ hopes too?

Several measures could be adopted to limit this issue: pharmaceutical companies could provide protocol amendments allowing more flexibility on timing and modality of protocol assessments without hampering treatment safety and efficacy; patients’ transfer to dedicated COVID-free research cancer centers to continue study treatments should be allowed. Furthermore, remote monitoring programs could be useful to maintain the supervision of clinical sites. Virtual visits could be implemented to reduce not necessary hospital visits. Biochemical exams could be performed in accredited facilities closer to patients’ residence. The risk/benefit ratio should be reassessed at each programmed hospital visit or treatment administration. National regulatory agencies are drawing up recommendations for clinical trials during COVID-19 pandemic [9].

Every effort should be made, both from clinicians and pharmaceutical companies, not to limit treatment chances for cancer patients and to concomitantly protect their safety.
